# Prospectively accelerated first-pass myocardial perfusion imaging in patients using motion-compensated compressed sensing exploiting regional low-rank sparsity

**DOI:** 10.1186/1532-429X-17-S1-O40

**Published:** 2015-02-03

**Authors:** Xiao Chen, Michael Salerno, Christopher M Kramer, Bhairav B Mehta, Yang Yang, Peter Shaw, Frederick H Epstein

**Affiliations:** 1 University of Virginia, Charlottesville, VA, USA

## Background

First-pass perfusion CMR utilizes accelerated imaging to achieve high spatial resolution and coverage within a small acquisition window. Several compressed sensing (CS) methods have been proposed to accelerate perfusion imaging^1-3^. However, patient motion due to imperfect breathholding and other factors leads to degraded quality of CS-reconstructed images. We recently demonstrated a CS method (Block LOw-rank Sparsity with Motion guidance, BLOSM^4^) that exploits regional low-rank sparsity and compensates for the effects of motion, and the dvantages of BLOSM were demonstrated using retrospectively-undersampled first-pass data^4^. In the present study, prospectively-accelerated first-pass data were collected from patients undergoing clinically ordered CMR studies, and we compared image quality for images reconstructed using BLOSM and the k-t SLR method^2^, a reference CS method that exploits global low-rank sparsity.

## Methods

Multislice 2D saturation-recovery first-pass gadolinium-enhanced data were collected from 10 patients on a 1.5T Avanto scanner using the standard body phased-array RF coil. For each patient, 3 short-axis slices were acquired per heartbeat for 50-70 heartbeats. A variable-density ky-t undersampling pattern following the poisson disk distribution was implemented to achieve an appropriate sampling pattern for CS reconstruction . With rate-4 acceleration, the acquisition window for one slice was 96 ms. Other parameters included: Cartesian trajectory, spatial resolution=1.8-2.1×1.8-2.1mm^2^, slice thickness=8mm, repetition time=2.4 ms, and saturation recovery time=100ms. The undersampled data were reconstructed using BLOSM and k-t SLR. Multi-coil data were combined using SENSE, with sensitivity maps calculated from temporally-averaged undersampled data. For a fair comparison, both BLOSM and k-t SLR were implemented using the same optimization algorithm and the reconstruction parameters were optimized for each method. Two cardiologists scored the overall image quality (scale of 1-5, where 1 is the best).

## Results

**Figure 1:**
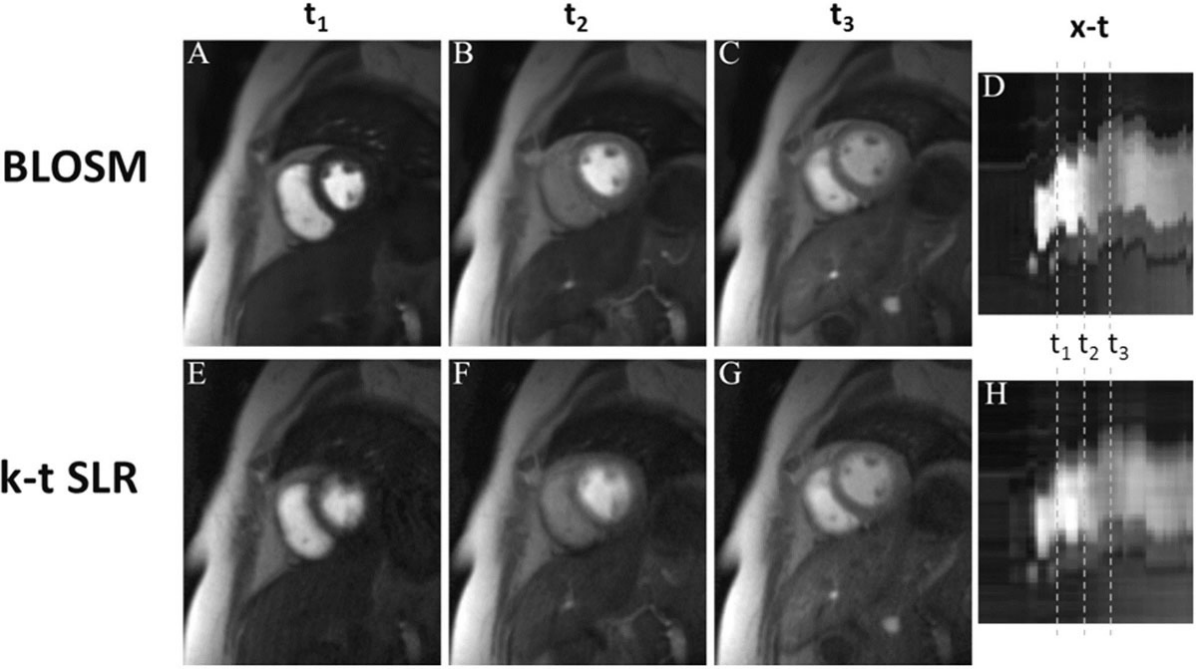
Example reconstruction results of one slice using BLOSM (A-D) and k-t SLR (F-I) from one patient at multiple time points. Images at three different time points (t_1_,t_2_,t_3_) and the corresponding spatial-temporal (x-t) profiles are shown in separate columns. The x-t profiles show that substantial respiratory motion occurred during the scan. BLOSM images demonstrate good motion compensation (A-C) whereas k-t SLR images suffered from blurring when motion occurred (t_1_, t_2_).

**Figure 2:**
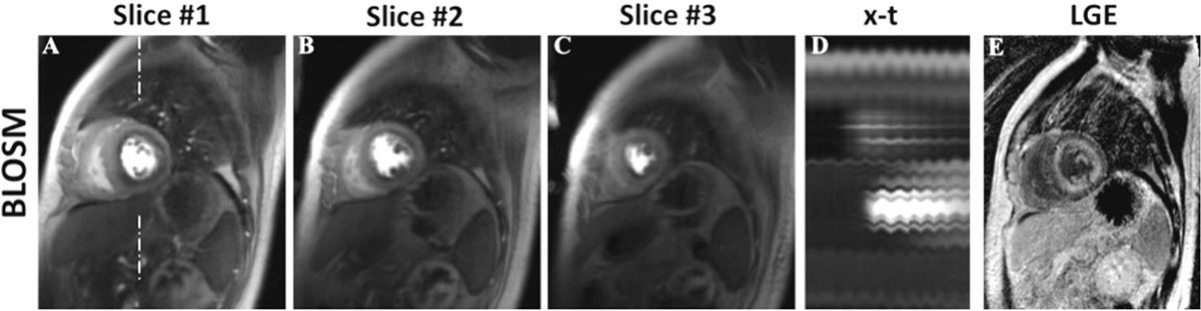
Example BLOSM reconstruction results from one patient with amyloidosis. Multi-slice images from one time point are shown (A-C), along with the x-t profile (D) and a corresponding LGE image (I). A subendocardial perfusion defect is clearly depicted by BLOSM, even in the presence of respiratory motion during the scan, as illustrated in the x-t profile. The subendocardial perfusion defect location matched closely with enhancement on the LGE image.

Figure 1 shows example BLOSM and k-t SLR reconstructed images from one slice at multiple time points. This example demonstrates that with prominent respiratory motion (see the x-t profiles in (D) and (H)), BLOSM (A-D) provides consistently good image quality, while k-t SLR (E-H) shows blurring (E,F). Figure 2 shows BLOSM results from three slices from a patient with a perfusion defect and prominent respiratory motion (D), along with a corresponding LGE image showing scar (E). Image quality scores were better for BLOSM (2.1±0.8 for BLOSM vs 2.9±0.7 for k-t SLR, p<0.01).

## Conclusions

High-quality prospectively-accelerated CS-reconstructed first-pass perfusion imaging was achieved in heart-disease patients using BLOSM, even when substantial respiratory motion occurred. These findings support the use of regional low-rank sparsity with motion compensation.

## Funding

This work was supported by NIH grants R01 EB 001763, R01 HL 115225, K23 HL112910, American Heart Association Predoctoral Award 12PRE12040059 and Siemens Medical Solutions.

## References

[B1] Otazo MRM 2010

[B2] Lingala IEEETMI 2011

[B3] Akcakaya MRM 2013

[B4] Chen MRM 2013

